# Post-antibiotic effect of orbifloxacin against *Escherichia coli *and *Pseudomonas aeruginosa *isolates from dogs

**DOI:** 10.1186/1751-0147-54-16

**Published:** 2012-03-20

**Authors:** Kazuki Harada, Takae Shimizu, Yasushi Kataoka, Toshio Takahashi

**Affiliations:** 1 Laboratory of Veterinary Microbiology, Nippon Veterinary and Life Science University, 1-7-1, Kyonan-cho, Musashino, Tokyo 180-8602, Japan

## Abstract

Orbifloxacin is a fluoroquinolone drug used widely in companion animal medicine. In this study, we firstly determined post-antibiotic effects (PAEs) and post-antibiotic sub-minimum inhibitory concentrations (MIC) effects (PA-SMEs) of orbifloxacin for two strains each of *Escherichia coli *and *Pseudomonas aeruginosa *from dogs, and these parameters were compared with those of enrofloxacin. At twice the MIC, the PAEs of orbifloxacin ranged from -0.28-0.93 h (mean, 0.29 h) for *E. coli *and -0.18-1.18 h (mean, 0.37 h) for *P. aeruginosa*. These parameters were not significantly different for *E. coli *and shorter for *P. aeruginosa*, compared to enrofloxacin (*P *< 0.05). Continued exposure to 0.1, 0.2, and 0.3 the MIC of orbifloxacin resulted in average PA-SMEs of 0.55, 1.11, and 2.03 h, respectively, for *E. coli*, and 1.04, 1.40, and 2.47 h, respectively, for *P. aeruginosa*. These PA-SMEs, which had no significant differences with those of enrofloxacin, were significantly longer than the corresponding PAEs (*P *< 0.05). These results suggest that the PA-SME of orbifloxacin for *E. coli *and *P. aeruginosa *can be meaningfully prolonged by increase of sub-MICs.

## Findings

Orbifloxacin is a fluoroquinolone developed for use in companion animal medicine. This antimicrobial agent exhibits bactericidal activity against numerous gram-negative and gram-positive bacteria. In canine practice, orbifloxacin is indicated for the treatment of various infections, including urinary, skin, and otitis infections, and is available in many countries including Japan.

Pharmacodynamic variables such as the post-antibiotic effect (PAE) and post-antibiotic sub-minimum inhibitory concentration effect (PA-SME) have increasingly become the focus of investigations designed to determine optimal dosage regimens for antimicrobial agents. The PAE is defined as the length of time that bacterial growth is suppressed following brief exposure to an antibiotic [1]. PAE has been investigated for several veterinary fluoroquinolones, such as enrofloxacin, marbofloxacin, and difloxacin [2-5], but not for orbifloxacin. On the other hand, PA-SME is defined as the time interval that includes the PAE plus the additional time during which growth is suppressed by sub-MICs, and has not been investigated for all veterinary fluoroquinolones, including orbifloxacin. In this study, we examined the *in vitro *PAEs and PA-SMEs of orbifloxacin against *Escherichia coli *and *Pseudomonas aeruginosa*, which are representative gram-negative pathogens responsible for urinary and skin infections, respectively, in dogs, and these values were compared with those for enrofloxacin.

Two strains each of *E. coli *(strains 09-207 and 09-225) and *P. aeruginosa *(strains 33 and 72) were used in this study. These organisms were isolated from canine urine (*E. coli*) and skin (*P. aeruginosa*), and identified by gram stain, catalase, and oxidase tests and Api 20E kit (Bio Merieux, France). MICs of orbifloxacin and enrofloxacin were determined by the agar dilution method according to the guidelines of the Clinical and Laboratory Standards Institute [6]. *E. coli *ATCC 25922 and *P. aeruginosa *ATCC 27853 were used as quality control strains.

The PAE and PA-SME of orbifloxacin were examined in comparison with enrofloxacin, and were performed in accordance with procedures described previously [7]. Each strain was grown in the logarithmic growth phase to a concentration of approximately 5 × 10^6 ^colony-forming units (CFU)/mL and was prepared for use in PAE experiments. One hour of exposure to orbifloxacin and enrofloxacin at twice the MIC reduced the starting inoculum by approximately 1-2 log_10 _units. Growth controls with inoculum but no antibiotic were included for each experiment. Tubes were placed in a shaking water bath at 35°C for 1 h. Following exposure to fluoroquinolones, bacteria were removed from the fluoroquinolones by pelleting the cells by centrifugation at appropriate conditions. The bacteria were resuspended in fresh, drug-free cation-adjusted Mueller-Hinton broth (CAMHB), once again pelleted by centrifugation, and resuspended again in fresh, drug-free CAMHB. Control organisms were not exposed to either fluoroquinolone but were treated similarly. Following drug removal, the fluoroquinolone-exposed and control cultures were placed in fresh media and incubated in a water bath at 35°C with agitation (100 rpm). Viability counts were determined before exposure, immediately after centrifugation (0 h), and then hourly for 5 h by plate counting. A test of final colony counts was performed at 24 h to allow for the sufficient growth of all samples. The PAE was defined according to the formula: PAE (in hours) = *T*-*C*, where *T *is the time required for viability counts of an antibiotic-exposed culture to increase by 1 log unit above counts taken immediately after dilution and *C *is the corresponding time for the growth control, as previously described [1].

**Figure 1:**
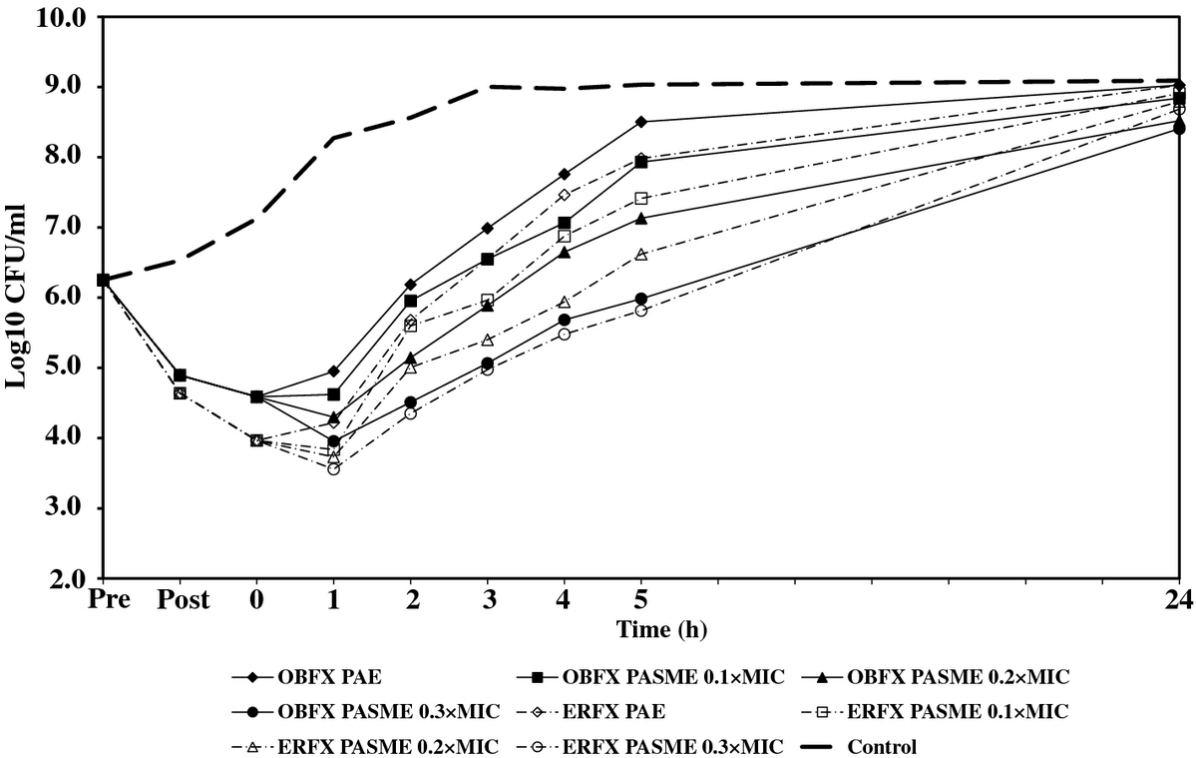
**PAE and PA-SME of orbifloxacin and enrofloxacin for *E. coli *09-207**. Pre: The time of beginning exposure to the fluoroquinolone at twice the MIC. Post: The time of discontinuing exposure to the fluoroquinolone at twice the MIC.

**Figure 2:**
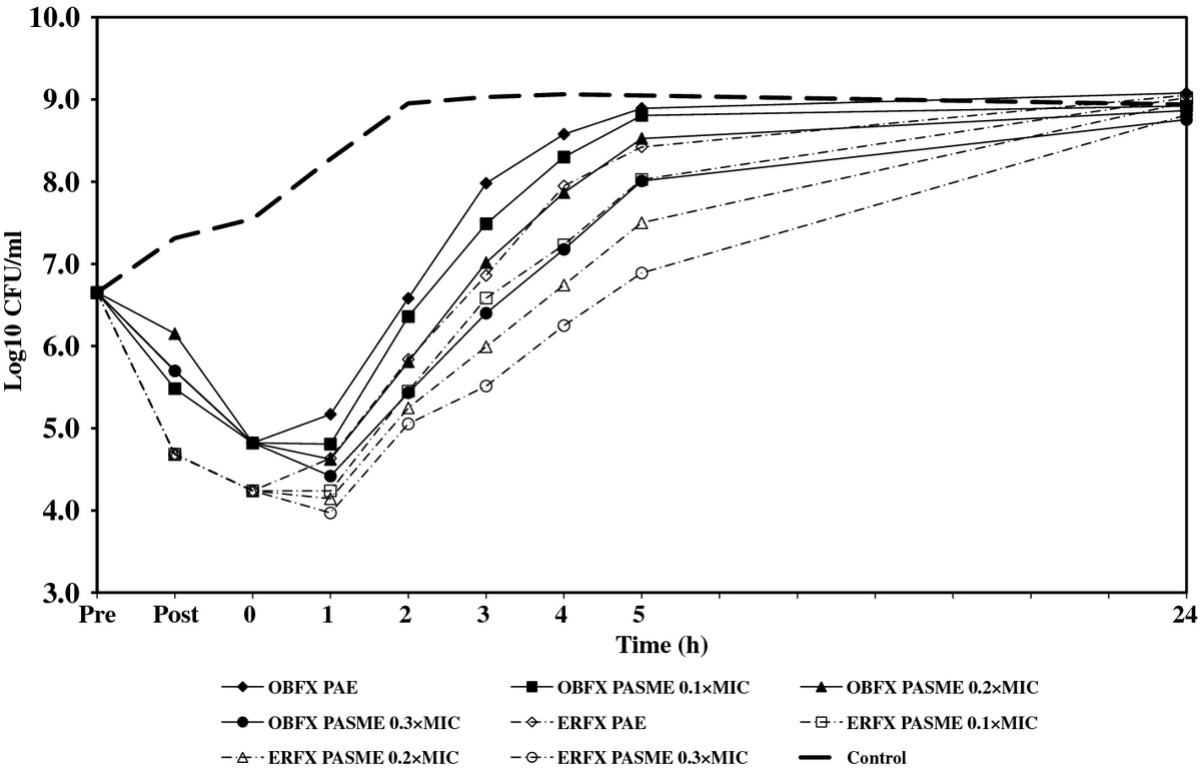
**PAE and PA-SME of orbifloxacin and enrofloxacin for *E. coli *09-225**. Pre: The time of beginning exposure to the fluoroquinolone at twice the MIC. Post: The time of discontinuing exposure to the fluoroquinolone at twice the MIC.

**Figure 3:**
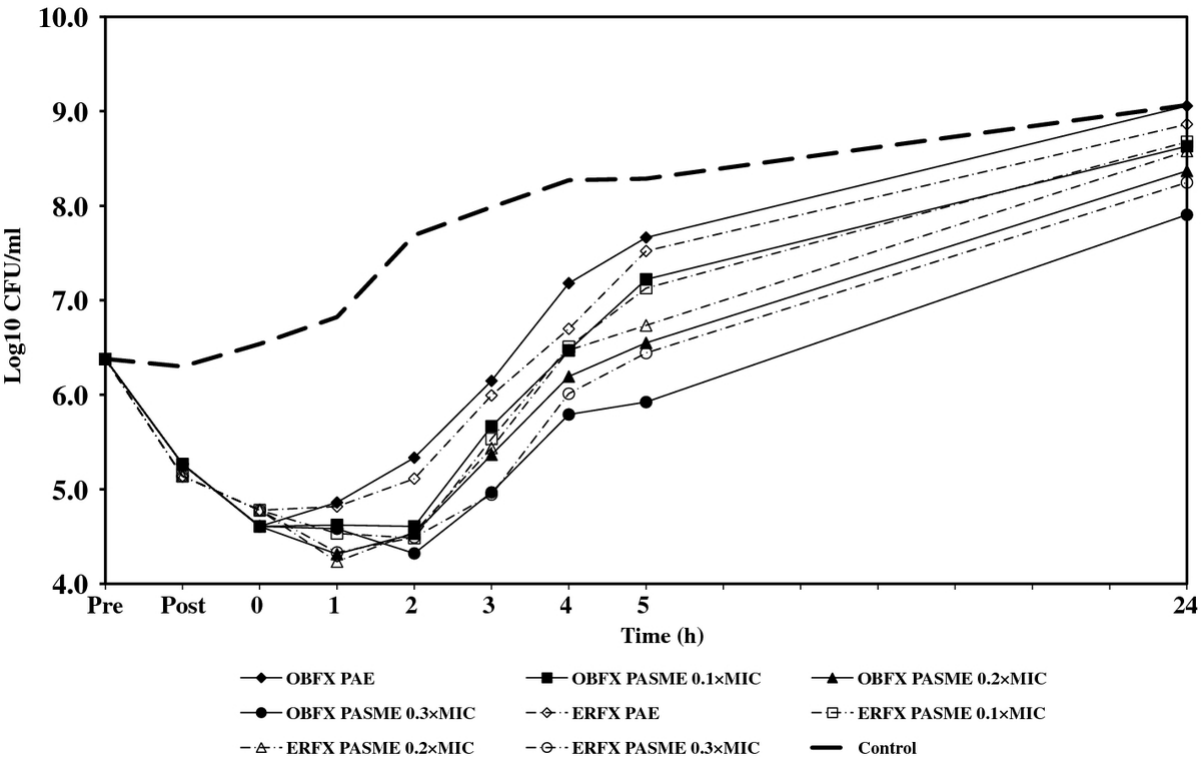
**PAE and PA-SME of orbifloxacin and enrofloxacin for *P. aeruginosa *72**. Pre: The time of beginning exposure to the fluoroquinolone at twice the MIC. Post: The time of discontinuing exposure to the fluoroquinolone at twice the MIC.

**Figure 4:**
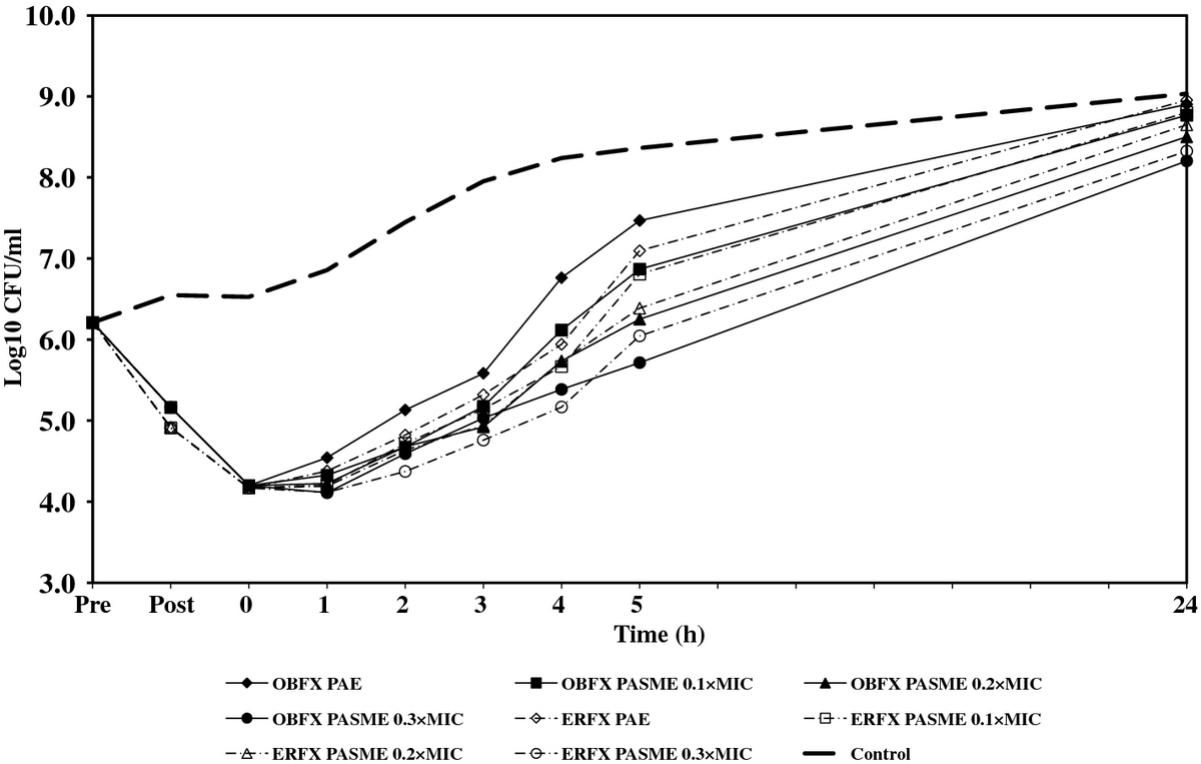
**PAE and PA-SME of orbifloxacin and enrofloxacin for *P. aeruginosa *33**. Pre: The time of beginning exposure to the fluoroquinolone at twice the MIC. Post: The time of discontinuing exposure to the fluoroquinolone at twice the MIC.

In cultures designated for PA-SME, the PA-phase *E. coli *or *P. aeruginosa *organisms were exposed to different sub-MICs (0.1, 0.2, and 0.3 times the MIC) of orbifloxacin and enrofloxacin. One sample of PA-phase bacteria to which no drug was added served as the control. All samples and controls were incubated in a water bath at 35°C with agitation (100 rpm) and the growth of all cultures was monitored by determining viable cell counts, as described above. The PA-SME was calculated using the equation: PA-SME (in hours) = *T_PA_-C*, where *T_PA _*is the time required for sub-MIC-treated PA-phase organisms to grow to 1 log unit and *C *is the time required for unexposed organisms to grow to 1 log unit, as previously described [1]. The PAE and PA-SME were measured in three independent experiments. The Student's *t *test was used to determine the significant differences (*P *< 0.05) between the two groups. For each experiment, viability counts (log CFU/mL) were plotted against time and expressed as the means of results from three separate assays. The fluctuations of bacterial numbers during experiments are shown in Figures 1, 2, 3, 4, and MICs, PAEs, and PA-SMEs are summarized in Table 1.

**Table 1:** PAEs and PA-SMEs of orbifloxacin and enrofloxacin for canine *E.coli *and *P. aeruginosa *isolates

Organism (origin)	Antibiotic	MIC (μg/ml)	PAE (h)^a, b^	PA SME (h)^b^		
				
				0.1 the MIC	0.2 the MIC	0.3 the MIC
*E. coli *09-207	Orbifloxacin	0.125	0.53 (0.12-0.93)	0.80 (0.54-1.27)	1.61 (1.31-2.00)	3.04 (2.68-3.70)
	Enrofloxacin	0.031	0.52 (-0.12-0.99)	0.76 (0.10-1.50)	1.15 (0.45-2.04)	1.93 (1.07-2.72)
*E. coli *09-225	Orbifloxacin	2	0.05 (-0.28-0.27)	0.29 (0.09-0.51)	0.61 (0.20-0.84)	1.02 (0.46-1.31)
	Enrofloxacin	0.5	0.13 (-0.11-0.54)	0.40 (-0.08-0.85)	0.60 (0.01-1.40)	0.84 (0.12-1.34)
*E. coli *(mean)	Orbifloxacin	-	0.29	0.55	1.11	2.03
	Enrofloxacin	-	0.32	0.58	0.87	1.38
*P. aeruginosa *72	Orbifloxacin	2	0.62 (0.25-1.18)	1.19 (0.59-1.81)	1.65 (0.86-2.23)	3.34 (1.34-6.14)
	Enrofloxacin	0.5	1.04 (0.79-1.39)	1.37 (1.08-1.81)	1.42 (1.25-1.66)	2.15 (1.37-2.71)
*P. aeruginosa *33	Orbifloxacin	4	0.13 (-0.18-0.50)	0.88 (0.55-1.78)	1.14 (0.54-1.90)	1.60 (0.64-2.27)
	Enrofloxacin	1	0.71 (0.05-1.53)	1.06 (0.31-1.95)	1.20 (0.43-2.14)	1.74 (1.07-2.74)
*P. aeruginosa *(mean)	Orbifloxacin	-	0.37	1.04	1.40	2.47
	Enrofloxacin	-	0.87	1.22	1.31	1.94

^a^Organisms exposed to two times the MIC.

^b^Averages of three individual experiments; ranges in parentheses.

In this study, the average PAE of orbifloxacin for *E. coli *was not significantly different from that of enrofloxacin (0.29 h vs. 0.32 h, *P *= 0.84), whereas the average PAE of orbifloxacin for *P. aeruginosa *was significantly shorter than that of enrofloxacin (0.37 h vs. 0.87 h, *P *= 0.022). In previous studies, van den Hoven *et al. *[3] reported that difloxacin had PAEs of 0.5 h for *E. coli *and -0.4 h for *P. aeruginosa*. Spreng *et al. *[2] reported PAEs for marbofloxacin that ranged from 0.6 to 0.9 h for *E. coli*. These PAEs were induced by the exposure of the drug at twice the MIC for 1 h, which is the same condition as in the present study. Thus, orbifloxacin is likely to have average PAEs for *E. coli *and *P. aeruginosa *among veterinary fluoroquinolone drugs.

The PA-SMEs have been studied extensively in most antibiotics with bacterial species in human medicine [1] but not in veterinary medicine. In this study, we first determined PA-SMEs of two veterinary fluoroquinolones, orbifloxacin and enrofloxacin. Following continued exposure to orbifloxacin at 0.1, 0.2, and 0.3 times the MIC, the average PA-SMEs were observed at 0.55, 1.11, and 2.03 h, respectively, for *E. coli*, and 1.04, 1.40, and 2.47 h, respectively, for *P. aeruginosa*. On the other hand, the PA-SMEs of enrofloxacin at each concentration were 0.58, 0.87, and 1.38 h, respectively, for *E. coli *and 1.22, 1.31, and 1.94 h, respectively, for *P. aeruginosa*. There were no significant differences in values of PA-SMEs between orbifloxacin and enrofloxacin (*P *≥ 0.17). The PA-SMEs of both drugs were significantly longer than the corresponding PAEs (*P *≤ 0.037), which may suggest that the PA-SME of orbifloxacin, as well as enrofloxacin, for *E. coli *and *P. aeruginosa *can be prolonged by increased sub-MICs.

The relationship between MIC and pharmacodynamic parameters, including PAE and PA-SME, is not entirely understood. This study showed that the strains with higher MICs of orbifloxacin and enrofloxacin (i.e. *E. coli *09-225 and *P. aeruginosa *33) consistently showed shorter PAE and PA-SME, compared with those with lower MICs (i.e. *E. coli *09-207 and *P. aeruginosa *72), respectively. Notably, there were significant differences in the PA-SMEs of orbifloxacin at 0.2 and 0.3 times the MIC between the two *E. coli *strains (*P *≤ 0.027). These results suggest that MICs can affect the periods of PAE and PA-SME in bacteria. However, Licata *et al. *[7] reported that the two strains of *Staphylococcus aureus*, with the same MICs, showed different PAEs and PA-SMEs. Therefore, the effect of MICs on PAEs and PA-SMEs in bacteria requires further investigation.

In conclusion, our study showed that orbifloxacin has meaningful PAEs and PA-SMEs for *E. coli *and *P. aeruginosa *isolates from dogs. The exposure concentrations of orbifloxacin to induce PAE and PA-SME in this study are achievable at each infection site (i.e., urine and skin) by usual therapeutic doses [8,9]. For these organisms, however, other important factors affecting antimicrobial potency (e.g. bactericidal effect [10] and postantibiotic leukocyte enhancement [11]) remain to be clarified. To additionally validate regimen of veterinary fluoroquinolones including orbifloxacin, further studies would be needed.

## Competing interests

This study was supported by a grant from DS Pharma Animal Health Co., Ltd., Japan. The sponsor of the study had no role in the study design, conduct of the study, data collection, data interpretation or preparing of the manuscript.

## Authors' contributions

KH and TS carried out all experiments and equally contributed to this study. KH was involved in the study design. KH, YK, and TT was preparation of the manuscript. KH drafted the manuscript. All authors read and approved the final manuscript.
